# Congenital Mucocele of the Nasal Dorsum: A Case Report

**DOI:** 10.4274/tao.2021.6209

**Published:** 2021-03-26

**Authors:** Seçil Bahar Dal, Ömer Faruk Ünal

**Affiliations:** 1 Department of Otorhinolaryngology, Vehbi Koç Foundation American Hospital, İstanbul, Turkey

**Keywords:** Nasal dorsum, mucocele, congenital, cyst, newborn, pediatric otorhinolaryngology

## Abstract

Congenital nasal dorsum cysts are very rare lesions. Its differential diagnosis lies between gliomas, dermoid cysts and encephaloceles. We present a case of solitary congenital external nasal cyst with no intranasal fistulous tract connection in a newborn. Histopathologic analysis of the mass demonstrated findings consistent with an external mucocele. Total excision with external open approach provided the cure with good cosmetic outcome. This is the first report presenting an external mucocele in a newborn in the literature. External mucoceles should be kept in mind in the differential diagnosis of congenital nasal dorsum masses.

## Introduction

Congenital nasal dorsal masses are rare, but mostly well-known pathologies. Differential diagnosis of these nasal lesions includes nasal dermoid cysts, gliomas and encephaloceles (1).

In this article we report the case of a newborn with a congenital external mucocele located on the tip of the nose. To the best of our knowledge, such a malformation in a newborn has not been described in the literature to date.

## Case Presentation

**Figure 1:**
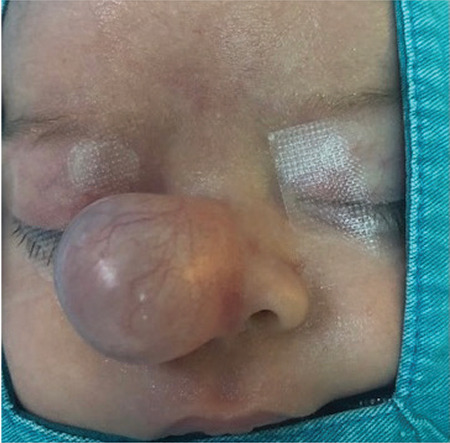
Preoperative view of the patient

A one-month-old male baby was observed to have a lesion on his nose at birth. The 2x2 cm, cystic and round shaped mass was located on the tip of the nose (Figure 1). 

**Figure 2:**
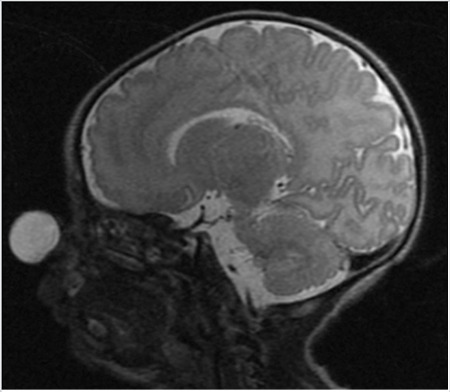
Sagittal T2 MRI view of the lesion MRI: Magnetic resonance imaging

Magnetic resonance imaging (MRI) showed a well-circumscribed, T2 hyperintense, homogeneous cystic mass on the tip of the nose with no evidence of any fistulous tract into the cranial or nasal cavity (Figure 2).

**Figure 3:**
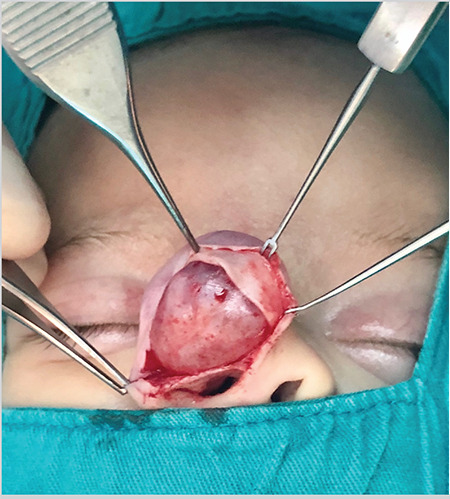
Intraoperative view of the nasal dorsal mass, showing the mucous cyst being dissected from the overlying skin

Direct open approach with an alar rim incision was used to remove the cyst. The cyst was just under the skin without any connection to the cartilages of the nasal dorsum (Figure 3). We did not observe any connection between the cyst and the nasal cavity during the operation.

**Figure 4:**
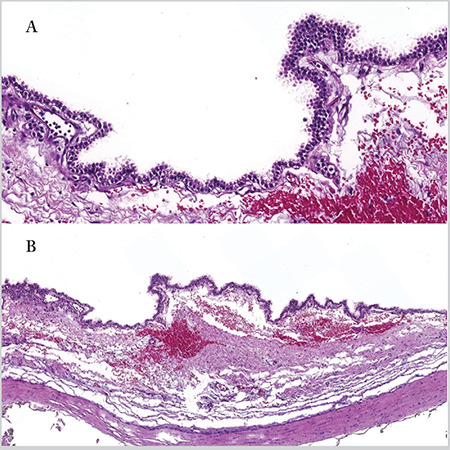
A, B. Histopathologic examination revealed an external mucocele, lined mainly with ciliated epithelium and the regions of focal pseudostratified columnar epithelium (Figure A x72 magnification with 3DHISTECH Case Viewer and Figure B x20 magnification with 3DHISTECH Case Viewer)

Histopathologic examination revealed an external mucocele composed mainly of ciliated epithelium and regions of focal pseudostratified columnar epithelium without lymphoid tissue, seromucous gland, goblet cell, or crypt. Pressure atrophy caused by fluid accumulation was observed in the epithelial lining of the cyst (Figure 4A,B).

**Figure 5:**
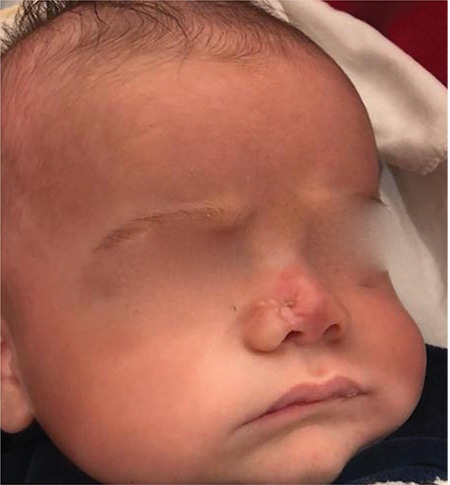
Postoperative first month view of the patient

The tip of the nose was packed for two days after the operation. There was no complication during or after the operation. The patient’s appearance one month after the operation is shown in Figure 5. We did not observe any recurrence in a six-month follow-up period.

Informed consent was taken from the father of the patient for publication.

## Discussion

Mucus retention cysts, benign skin adnexal tumors, cholesterol granulomas, dermoid and epidermoid cysts and encephalocele are included  in the differential diagnosis of a nasal mucocele (2, 3, 4).

After detailed histopathological examination, presented case was diagnosed as an external mucocele. Histopathologically, the most similar lesions to our case are the cyst formations following rhinoplasty. The sites of these cysts vary from the glabellar region to the tip of the nose and the paranasal sinuses. Herniation of the mucosa, interposition or inoculation of the nasal mucosa are supposed to be the most accepted explanations of this entity (3, 5).

Similarly, in our case, proliferation of the cells in an ectopic mucous membrane island seems to be an acceptable theory of etiology. Occlusion of sebaceous glands, as reported by Rettinger and Steininger (6), can also lead to such mucous cysts.

Comprehensive physical examination is important for diagnosis. Also, preoperative imaging of nasal congenital lesions is an essential tool to confirm the diagnosis. It is important to bear in mind intracranial extension of intranasal masses. Understanding the extent of the lesion with MRI will help to tailor the surgical approach, hence completely excise the lesion and prevent recurrences. In this type of lesions, it is also important to remove the cyst and the fistulous pathway in the same session to avoid possible infectious complications (1, 7).

Given the risks of exposure to radiation, computed tomography scanning is not always necessary for diagnosis in young children, as in our case, when MRI findings provide adequate preoperative information. In patients with uncertain MRI findings or when further bone anatomy evaluation is needed, computed tomography scan can also be performed (7). MRI findings were consistent with intraoperative findings and we did not observe any fistulous tract extensions of the cystic mass in the presented case.

The treatment of choice is complete excision with intact capsule. For similar nasal masses, open or closed rhinoplasty approach, endoscopic excision or direct excision with external skin incision can be preferred (3, 7). Considering the location of the cyst we preferred a direct external open approach, which provided a wide exposure allowing a safe surgical excision and a relatively good aesthetic result.

**Main Points**

• Nasal dorsum cyst in a newborn is a rare lesion.

• Nasal dermoid and epidermoid cysts, gliomas, encephaloceles, benign skin adnexal tumor and cholesterol granulomas are well-known lesions in differential diagnosis.

• In patients with congenital nasal masses preoperative radiologic examination is crucial for differential diagnosis and for determining the extension of the lesion and the appropriate surgical planning.

• External mucoceles of the nasal dorsum are cyst formations that have been reported following rhinoplasty. However, external mucocele formations in the nasal dorsum can also occur as congenital lesions.
